# Quasi-Psychologism about Collective Intention

**DOI:** 10.1007/s10677-021-10188-2

**Published:** 2021-04-26

**Authors:** Matthew Rachar

**Affiliations:** Department of Philosophy, Faculty of Philosophy and Education, University of Vienna, Universitätsstraße 7, A-1010 Vienna, Austria

**Keywords:** collective intention, shared agency, collective action, joint intention, interpersonal obligation, sociality, experimental philosophy, positive program

## Abstract

This paper argues that a class of popular views of collective intention, which I call “quasi-psychologism”, faces a problem explaining common intuitions about collective action. Views in this class hold that collective intentions are realized in or constituted by individual, mental, participatory intentions. I argue that this metaphysical commitment entails persistence conditions that are in tension with a purported obligation to notify co-actors before leaving a collective action attested to by participants in experimental research about the interpersonal normativity of collective action. I then explore the possibilities open to quasi-psychologists for responding to this research.

Human beings have a remarkable capacity to purposefully do things together with others. Some think this capacity relies on another, our ability to form collective intentions in some way distinct from our individual intentions. The thought is that collective intentions undergird collective actions by coordinating across people and time in ways that other forms of intersubjectivity don’t. This thought naturally leads to three questions: (1) what kinds of things are collective intentions, if collectives don’t have minds and intentions are normally mental states? (2) how do our individual mental states stand to the collective intentions we form when we act together? And (3) what relations do collective intentions require between the people who have them?

This paper begins by laying out the most relevant answers to these questions along two lines, metaphysical and normative, and distinguishing between some relevant possible views. I then present some results from experimental research about the nature of adult human collective action and consider what role such research might play in philosophical reflection on the topic. One result in particular, which suggests that there are certain minimal interpersonal obligations in collective action, may cause a problem for a popular class of views, which I label “quasi-psychologism”. In addition to exploring the conceptual resources available to three quasi-psychological views for explaining these results, I evaluate the quasi-psychological project in general.

## The Metaphysical and Normative Axes of Collective Intention

The metaphysical axis concerns the strength of connection between individual psychological states and collective intentions. Quasi-psychological views hold that collective intentions are constituted by the individual intentions of the participants.^1^ They may be contrasted with, on the one hand, psychological views, which hold that collective intentions are a special kind of intention had by individuals, and on the other hand, with non-psychological views, which hold that collective intentions need not bear any relation to the individual intentions of the participants.^2^ Only on psychological views are collective intentions mental states. On quasi-psychological views and non-psychological views, they are states of affairs. But on quasi-psychological views, theses states of affairs are inextricably linked to particular mental states, while on non-psychological views, they aren’t.

Quasi-psychological views are initially plausible since they explain two central features of collective intentions, the interdependence they create between participants and their motivational force. These features are often taken to be unaccounted for by the other kinds of views. Many think that identifying collective intentions with psychological states of individuals precludes psychological views from capturing the causal and cognitive interdependence between participants in a collective action.^3^ Equally widespread is the claim that without positing a definite relation to individual intentions, collective intentions on non-psychological views are unable to explain how the individuals involved end up doing anything to execute the collective action.^4^ By rejecting the identification of collective intentions with individually held intentions, quasi-psychological views avoid the interdependence problem of psychological views. By maintaining a strong connection between individual intentions and collective intentions, they avoid the motivational problem of non-psychological views. These considerations have led to broad acceptance of quasi-psychologism.

What’s the problem with quasi-psychological views? Here I’ll argue that some recent empirical results, presented in the next section, may be one. These results cast doubt on the ability of quasi-psychological views to fulfil the purpose of introducing collective intention in the first place, namely explaining collective action. It appears they fail to provide the right background to properly characterize the normative relations between people who are acting together.

This brings us to the second axis, on which we may draw a distinction between normativist and non-normativist views.^5^ Normativist views hold that collective intentions constitutively or inherently involve normative relations, such as obligations, rights, and entitlements. Collective intentions are essentially, or essentially related to, social commitments, which involve mutual directed obligations between the participants. Non-normativist views deny this claim, and instead treat collective intention as a social-psychological structure, which may or may not be accompanied by normative relations.

Since the issue raised by the empirical research is about the interpersonal obligations in collective action, an intuitive thought is that a normative version of quasi-psychologism will generate a satisfactory response. Surprisingly, as I show in section 4, this turns out not to be the case. The normative quasi-psychological view I consider there, belonging to Abraham Roth, actually fares worse than some non-normativist versions of quasi-psychologism. But there is a way forward. Recently, Facundo Alonso has proposed a “dual-aspect” view, which intimately connects the social-psychological structure of collective intention with obligation-generating normative principles, without treating these relations as constitutive of collective intention. For Alonso, collective intention involves reliance, and since intentionally or negligently generating or reinforcing reliance creates obligations, forming a collective intention is, normally, an obligation-generating phenomenon. For quasi-psychologism, this strategy is best placed to explain the empirical results. Nonetheless, Alonso’s specific view still faces difficulties explaining the empirical research in two cases – minimal collective action and morally wrong collective action.

So, I argue that there is uncompleted work for quasi-psychologists about collective intention. If they are aiming for their theory to be sensitive to our social practices, they need either to incorporate some new tools from normative theory in order to explain the experimental results or to explain away these results as adjacent parts of our social environment or artefacts of the research itself. In the next section, I’ll discuss the empirical research concerning our social practices surrounding collective action.

## Experimental Research: Justification and Results

A new experimental paradigm explores and interrogates the descriptive and observational components of the philosophical work on collective intention and action.^6^ Many theorists of collective intention employ thought experiments about cases of acting together to elicit intuitions in support of a particular view. Some are explicit about this methodology. Margaret Gilbert, for example, claims that we should start our investigations “with observations on the way people think and talk about shared intention in everyday life”^7^ and suggests that “warrant for the description [of our common understanding of collective action] is informal observation including self-observation.”^8^ Even those who do not fully endorse that approach think that examining intuitions gives us some direction. About his own view, Michael Bratman says “I do think that the model I am sketching broadly coheres with pre-analytic talk of shared intention and of shared intentional and shared cooperative activities,” adding that “such pre-analytic talk can be a useful, if defeasible, guide”.^9^

On several issues, this intuitional methodology has led to stark disagreement. For example, the case for non-normativism, as Michael Bratman presents it, relies in part on examples of collective action that do not involve interpersonal obligations. Here is one Bratman takes to be a counterexample to the normativist thesis^10^:

Suppose you and I are independently walking down Fifth Avenue. We spot each other on 65^th^ Street, and we briefly walk together, chatting, until, as it happens, you peel off at 59^th^ Street. We do not merely walk individually along the same stretch of street and at the same time. Rather, we intentionally walk together for this brief time, and we briefly have a shared intention to walk together. Nevertheless, it seems strained to insist, baring the introduction of further features to the story, that either has an obligation to the other…

Gilbert, a leading normativist, tells a very similar story but draws a radically different conclusion:

Suppose that Heinrich and Andrea are going for a walk together…Suppose now that Heinrich suddenly claps his hand to his brow, says “Oh No!” and, without further ado, starts walking rapidly away from Andrea…Barring special background understandings, however, she will understand that – to put it somewhat abstractly – the manner of his going involved *a mistake*…Given that this is so, Andrea evidently understands that by virtue of their walking together she has a *right* of some kind to Heinrich’s continuing to walk alongside her…together with the standing to issue related rebukes and demands.^11^

From comparable stories about going for a walk, Bratman and Gilbert come to opposite views about fundamental aspects of acting together.^12^ For Bratman, the fact that minimal cases of acting together are possible even when it seems strained to attribute obligations and entitlements to the participants shows that there are at least some collective actions without normative relations. For Gilbert, the fact that *even in* such cases there are normative relations suggests that normative relations are essential to collective action.^13^

Given that these methods have had little success in leading philosophers to converge on a view or in offering plausible procedures for adjudicating between competing intuitions, there is reason to turn to the methods of experimental philosophy in order to make this method more precise. There are several benefits of doing so. First, it provides a reproducible procedure for arriving at a conclusion, something which is lacking in traditional thought experiments. Second, it allows for a more general understanding of the phenomenon, since by conducting the experiments with a sample larger than one, we gain assurance that particular conclusions are not the result of idiosyncratic personal factors based on differences in experience and background. Third, by systematically working through examples from several different sources and developing the vignettes in such a way that particular cues and behaviours are highlighted and tested, we come to a more detailed picture of our common understanding of collective action and its attendant interpersonal normativity. The guiding assumption is that this more detailed picture will provide clues about the suitability of various philosophical theories of collective intention, on the further assumption that they are aimed at explaining forms of human sociality.^14^

The empirical research discussed here extends the philosophical methodology by modifying thought experiments from the literature to develop experiments designed according to the methods of social psychology. For example, Gomez-Lavin & Rachar have developed several studies that assign participants to one of three conditions that manipulate the strength of evidence that the characters are walking together, based on the Bratmanian thought experiment quoted above.^15^ In the control condition (i.e. no collective action), participants read a prompt where two people are independently walking down the street beside each other from 65th street until 59th street on 5th Avenue, when one of them peels off. In collective action conditions, they walk together the same distance, but with the behavioural cues mentioned by Bratman, for example laughing and chatting, and again at 59th street one character suddenly leaves. Participants are asked a series of questions about the extent to which the characters are together and the appropriateness of, for example, leaving the collective action without notifying the other character. The results show a significant difference between cases involving collective action and those which don’t on both questions. More advanced statistical analysis strongly suggests that judgments that people are performing some action together are strongly correlated with judgments of interpersonal obligation, even in minimal cases.^16^

In a subsequent paper, Gomez-Lavin and Rachar added a fourth condition in which the characters promise one another that they will go for a walk and a new question about whether one character is required to seek the permission of the other before leaving.^17^ In addition to replicating the earlier results with respect to the original conditions and questions, these more recent studies suggest judgments about an “obligation to seek permission” from co-actors before leaving do not track judgments of togetherness. Instead, they appear to be associated with the specific act of promising, as many theorists of promissory obligation would expect.

Finally, Gomez-Lavin and Rachar tested our attribution of obligation in cases of immoral collective action. In the control condition, two people are lined up at an ATM booth.^18^ The machine malfunctions and starts dispensing $20 bills. One person begins collecting them, while the other person catches the few solitary bills and walks off. In the collective action condition, two people are actively breaking into an empty ATM booth late at night. One man has a crowbar and is using it to get into the ATM; the other man is waiting beside him with a bag to collect the cash. Before they get into it, the bagman suddenly walks out of the booth. Again, participants are asked about the togetherness of the characters and the appropriateness of leaving without notifying one’s co-actor, and again there is significant evidence of an association between the two. They also judged the action undertaken by the characters in the collective action vignette to be immoral.

For the purposes of this paper, I simplify and extrapolate from that research, creating a list of features of collective action that would best explain the results. This list isn’t definitive, especially since the experimental research on this topic is in early stages. Instead, these are motivated assumptions about the proper characterization of the interpersonal normative relations involved in collective action according to our common understanding of it.

The results of the studies conducted so far suggest that common intuitions about acting together in a strong sense affirm three judgments.:

(i) exiting a collective action involves an obligation to notify co-actors,
(ii) exiting a collective action does not involve an obligation to seek the permission of the co-actors.
(iii) the obligation in (i) is present in “morally wrong” cases of collective action.

It is certainly not the case that the experimental research is conclusive, nor could it provide a ground for or justification of these judgments. People do not have obligations in these contexts because the participants judged it so. But this research does tell us something about how a population conceives of the practices surrounding collective action, an issue that is important to philosophical work on the topic, in so far as the philosophical accounts in question are meant to capture something about actual human practices. And it is important to know whether such practices are in fact justified, a task that can only be completed by engaging in non-empirical, normative theorizing. Methodologically then, this research could play one of several roles in philosophical reflection. It could, inter alia, support a philosophical position by showing that the practices it justifies are our actual practices, prompt further normative theorizing by suggesting that there is a mismatch between the justifications offered by philosophers  and the practices as we find them in the world, or put pressure on philosophers to find some other way to explain the results that conflict with the aspects of their theories that they have argued for in part using intuitional methods.

In the next several sections, I’ll consider how well various quasi-psychological views fare explaining these results, and claim that, assuming they accept that these results have some role to play in theory selection, quasi-psychologists need to adopt new normative frameworks or incorporate as-yet-exploited tools in order to make sense of these social practices within the confines of the metaphysical commitments of their view.

## A Quasi-Psychological, Non-Normative View

The most prominent version of a quasi-psychological view of collective intention belongs to Michael Bratman. In this section, I highlight the metaphysical and normative structure of his view and consider its ability to explain the empirical results. Given the apparently normative nature of (i) and (iii), it may seem like non-normativist views are non-starters, but I point out how Bratman’s view is carefully constructed so as to be able to provide an explanation of these judgments without building normativity into the analysis. Nonetheless, I claim that the explanation his view is capable of providing gets the nature of the normativity in collective action wrong because it doesn’t adequately account for its content or structure.

For Bratman, “…a shared intention consists in a public, interlocking web of appropriate intentions of the individuals” identifiable by the roles they play in coordinating our social lives^19^ Roughly, Bratman argues that this web consists in individual participatory intentions towards the joint activity on the part of each co-actor, a further intention that the others’ intentions are effective, again for each co-actor, and common knowledge that all these intentions are in place. This structure of interrelated intentions and beliefs creates a shared practical commitment between the parties that puts rational pressure on them to coordinate their actions and practical reasoning in ways that reliably guide them towards the achievement of their aim, where the source of the rational pressure is the set of norms associated with intention, allowing shared intention to play several characteristic roles in human coordination.^20^

Bratman’s is clearly a quasi-psychological view, and it is just as clearly non-normative, with respect to the interpersonal normativity at issue here. As discussed above, he thinks that there are some collective actions – in the sense of things done with a shared intention to do them – that do not involve mutual obligation. While he acknowledges that “mutual obligations and entitlements are extremely common in cases of modest sociality”, he is “not convinced that such obligations are essential to modest sociality” and is convinced “that these will normally be familiar kinds of moral obligation – in particular, moral obligations associated with assurance, reliance, promises, and the like.”^21^

Given this, it makes sense to consider the following question: can Bratman’s theory deal with the presence of an obligation to notify in collective action? On his behalf, one might argue that the participants’ judgments are in fact about the rational requirements of shared practical commitments. In that case, the “obligation” to notify is really a rational requirement based on the norms that govern intentions. One of the requirements does stand out as a potential source for this “obligation,” namely the norm of stability, which says that, while subject to reconsideration, intentions should be stable over time. In some places, this is what Bratman suggests for similar situations.^22^ He argues that there is rational and social pressure towards stability that demands that agents engaging in shared agency do not too easily abandon their participatory intentions for two reasons. One is that reconsideration of a previously formed intention can have significant costs. The other, which is especially relevant here, is that it undermines coordination. An agent who too easily reconsiders her prior intentions will be a less reliable partner in social coordination. Because people avoid partnering with unreliable partners, she will end up with fewer opportunities to reap the benefits of that coordination. For Bratman, when an agent does too easily abandon a participatory intention, she is being *unreasonable*.^23^ And so, the story might go, the participants’ reactions are actually tracking the unreasonableness of the person for recklessly abandoning their intention, as represented by leaving without saying anything, something that would be salient to them given the negative consequences for social coordination more generally.

This story doesn’t work for two reasons. First, it doesn’t explain why, in a collective action, one actor owes the other something.^24^ The leaver doesn’t just have an obligation *simpliciter*; the leaver is obligated *to the leavee.* The obligation to notify is directed; the demands of rationality on intention are not. On Bratman’s view of rationality and planning agency, the demands of rationality are given in part by the norms mentioned above, which gain their normative significance from their ability to support a substantive value, self-governance.^25^ But this value is inherently self-regarding. It is not well-placed to explain interpersonal normative relations, and so it is not well-placed to explain why the leaver owes the leavee anything. At best, it could explain why planning agents owe it to themselves to have stable intentions, assuming that agents capable of being planning agents have an obligation to be planning agents. Even this, though, does injustice to the way Bratman talks about the value of self-governance and the norms of intention, since it suggests a specific, self-directed obligation rather than a general demand of rationality.

Second, Bratman’s potential unreasonableness answer requires that the participatory intention is dropped recklessly, or at least, without the proper process of reconsideration. But this isn’t specified by the story, either as told by Bratman or as told in the vignettes. The leaver may have reconsidered her participatory intention and come to the conclusion to drop it through rational deliberation. It may be entirely reasonable for the leaver to leave. In that case, the leaver satisfies the demand given by the norm of stability. Given that in the vignette nothing about the leaver’s practical reasoning is mentioned, there is no motivation to assume that the participants’ judgments about the obligation the leaver violates are based on a further judgment that the leaver’s participatory intention is irrationally discarded.

Another potential Bratmanian response focuses on his idea that moral obligations are often present in cases of collective action, even though they aren’t necessary. The reason to think this is that the etiology or maintenance of a collective intention commonly involves an obligation-generating process, the exchange of mutual assurances.^26^ According to Thomas Scanlon, on whose work Bratman is here relying, voluntarily and intentionally leading someone else to expect that you will perform some action is assuring them you will perform that action. And assuring someone that you will perform an action, for example by explicitly promising, generates an obligation to perform that action. Since acting together is a context in which we often voluntarily and intentionally induce expectations in others about our own behavior, it is a context in which there are frequently moral obligations.

Scanlon specifies the conditions under which assurance generate obligations and the content of those obligations in what he calls *Principle F*.^27^ Bratman holds that this principle, in conjunction with the fact that mutual assurances are often present in the formation or maintenance of shared intentions, explains why moral obligations are normal in collective actions.^28^ When acting with others, we want to be sure that the expectations we form about what everyone involved is going to do are actually going to be fulfilled. Adhering to *Principle F* is a way to satisfy this desire.

The interesting thing for our purposes is the way that Scanlon’s principle might generate an obligation to notify. In fact, it generates much more. One of the key features of *Principle F* is that it grants obligations of strict performance, which require the consent of the obligee for faultless breach. So, if *Principle F* governs the normativity of collective action, we would expect not only an obligation to notify before leaving in the walking case, but also an obligation to seek the other participants’ permission.^29^ This obligation is much stronger than the obligation to notify, stronger than appears appropriate according to our common understanding of collective action. That our social practices do not include an obligation to seek the permission of the other participants to leave, unless there has been an explicit exchange of promises, is the second result of the experimental research. And skepticism towards the presence of this obligation is common in the philosophical literature as well. Bratman, for one, is very much aware of cases of collective action that do not involve an obligation to seek permission, cases in which expectations are not purposely created. They are precisely the cases he proposes as counterexamples to the normativist thesis.

So, the second Bratmanian strategy fails to explain the putative obligation to notify in the walking case. By construction, Bratman’s walking case does not involve the intentional and voluntary creation of expectations. This seems like the right analysis of the experimental research vignettes. Excluding the promise conditions, no mutual assurances are exchanged, and so there is no moral obligations generated by *Principle F*. But, because *Principle F* doesn’t apply, and he doesn’t suggest another source of obligation, it is unclear on Bratman’s picture how the obligation to notify arises.

Providing a suitable explanation of the obligation to notify requires a different normative principle than the one on which Bratman relies. So, let’s turn to normativist views to see whether they can offer an alternative.

## A Quasi-Psychological, Normative View

Abraham Roth develops a quasi-psychological, normative view. When two or more people share an intention, it is constituted by each having a personal participatory intention and a special relation existing between the intentions, a special relation described in more detail below. What distinguishes Roth’s view from Bratman’s is that he thinks this psychological structure is inherently and interpersonally normative.

The intuitions in support of this claim are based on a walking case, originally presented by Gilbert, which is importantly different from the one discussed above. In the version of Gilbert’s example Roth discusses, Jack and Sue are going for a walk. Jack starts to pull ahead, walking too quickly for Sue to keep up. Gilbert claims that Sue is entitled to criticize Jack, and this suggests that Jack has an obligation to Sue.^30^ Roth endorses this picture, calling this kinds of obligations *contralateral commitments.*^31^ He also clarifies their nature, holding that they are *special* and *executive*. To be special is to hold only between participants in the collective action. To be executive is to treat any criticism the commitment licenses as part of the activity itself. Neither of these things apply to a third parties’ comments.

Roth maintains both normativism and quasi-psychologism by denying that agents can be motivated in their deliberation and action only by their own psychological states. Instead, shared intention is based on a relation between individuals that makes it possible “for one individual to take up and act on an intention formed by another without re-issuing the latter’s intention”, which Roth calls *practical intimacy*.^32^ In other words, if *A* is aware of *B*’s intention that *A* perform action *φ*, and practical intimacy exists between *A* and *B*, then *A* can “act directly” on *B*’s intention that *A φ*. Importantly, when *A φ*’s, they do so intentionally and the intention must be communicated from *B* to *A* so that *A* has knowledge or is aware of the intention. There is no “action at a distance.”^33^ And in acting on *B*’s intention for *A* to *φ*, *A* also intends to *φ*. But, because the intention to *φ* isn’t arrived at by a piece of practical reasoning on *A*’s part, it isn’t *A*’s intention. The key point is that acting directly on an intention means not having to re-issue that intention. In the normal case, if someone asks me to do something, I deliberate about whether to do it, and then form my own intention if I decide to. That is re-issuing the intention. But when practical intimacy exists between us, I don’t have to re-issue your intention, I can act on it by *preserving and executing* it, where this doesn’t involve me deliberating about what to do.^34^

According to Roth, practical intimacy generates contralateral commitments.^35^ Jack and Sue have the commitments they do because of the relation between their intentions. Sue has the intention that Jack walk with her. Jack is acting directly on that intention, and so, Sue, as the “owner” of the intention, has some license to direct Jack’s carrying out of the action. Jack is committed to Sue to follow through on the intention in the way Sue specifies. If he doesn’t, he lets her down. Further, Sue has a reciprocal commitment to Jack. Even though Jack is not the “owner” of the intention, he is bypassing the normal exercise of his rational agency in adopting it. He therefore has an entitlement to Sue’s intention as a practical commitment about what to do that matches his entitlement to his own personal intentions. So, Sue has special responsibilities not to undermine the practical conclusions upon which Jack is acting, for example by acting in ways that preclude his carrying out of her intention.^36^ Jack’s commitment to Sue and Sue’s commitment to Jack are then both special, since they hold as a result of the practical intimacy between them, which does not exist for third parties, and executive, since any criticism they make of each other is part of their carrying out of her intention.

But the set of normative relations Roth’s account accommodates doesn’t include the obligation to notify. The difference between the cases, Heinrich and Andrea in the experimental research, and Jack and Sue in Roth’s exposition, gives us a clue why. Heinrich suddenly walks away from the activity, ending it, but Jack doesn’t. He is still in the middle of the walk; he’s just doing it wrong. Jack and Sue both still have participatory intentions toward their action, and, according to Roth, it is this relation that matters. It governs the way the collective action should be carried out, given that everyone still intends to complete it. But that is not the case for Heinrich. Heinrich has already decided that he isn’t going to complete the action. Since he no longer has a participatory intention, there is no longer any practical intimacy. In turn, there is no contralateral commitment, which makes it unclear what could ground his obligation on Roth’s view.

The problem is particularly stark here, because of the way Roth conceives of the relation between *being committed* and *having a commitment*. For him, a participant to a collective intention has an interpersonal commitment only when they have an individual psychological commitment. This requirement stems from the fact that, for Roth, participatory individual commitment is the link through which intentions can be transferred between the participants. Jack preserves and executes Sue’s intention, rather than re-issuing it, in part because he has a participatory intention towards the action. But, as we have seen, being committed, in the sense of having a participatory intention, which is the sense Roth is invoking here, is open to reconsideration. The norm of stability tells us not to reconsider recklessly, but sometimes there is good reason to reconsider. What happens when Jack reconsiders, changes his mind, and no longer has that participatory intention?

The first thing that happens is that Jack is free to make Sue’s intention that he do something the object of his own rational faculties. Reflecting on Sue’s intention once he no longer intends to go for a walk may lead Jack, through rational steps, to conclude that he should drop Sue’s intention that he walk with her. This is open to him because as soon as he starts to deliberate about Sue’s intention, practical intimacy is broken. As long as Jack is acting on Sue’s intention, she has some authority over how he carries it out, but she doesn’t have any authority over *whether* Jack acts on it. His participatory intention is up to him because the use of his own deliberative faculties is up to him. And so, whether practical intimacy exists between them is up to him as well.

Since practical intimacy is dependent on Jack’s participatory intention in this way, contralateral commitments can’t explain the obligation to notify. Jack’s obligation to notify comes into effect only once he no longer has his participatory intention. While contralateral commitments provide a story about Jack’s obligation to walk at a reasonable pace as long as he has a participatory intention, they do not speak to his (or Heinrich’s) obligation to notify after he has decided he’s done with the walk. What’s required to explain the obligation to notify are normative commitments that outlive the participant’s psychological commitments. And this is ruled out by the structure of Roth’s view, since, for him, the presence of the normative commitments is dependent on the presence of the psychological ones.

The point about the existence of a collective intention generalizes to all quasi-psychological views. Since, on these views, collective intentions are combinations of individual mental states, their existence is dependent on all of those individual states being present. Once one of the participants drops their participatory intention, one of the necessary individual states is gone, and so there is no collective intention. And this is exactly the circumstance in which the obligation to notify comes into effect.

Surprisingly, the strategy of treating the quasi-psychological collective intentions themselves as the things that ground the interpersonal obligations of collective action actually takes such views farther away from being able to explain our common understanding of the interpersonal normativity of collective action.

## The Way Forward - a Dual-Aspect View

Normativism appears to be a wrong turn for quasi-psychological views. Our previous discussion suggests that the formation of a collective intention must trigger interpersonal obligations through some mechanism that does not require the continued existence of individual participatory intentions. What we need is a view that makes forming a collective intention an obligation-generating phenomenon without making those obligations dependent on the collective intention itself.

Such a view has been proposed by Facundo Alonso.^37^ Alonso adopts much of the Bratmanian structure with respect to the socio-psychological nature of collective intention – they are combinations of attitudes including individual participatory intentions toward the collective activity that play certain characteristic roles - with one crucial substitution. Instead of beliefs about the others’ intentions, Alonso argues that we come to *rely* on our fellow co-actors’ intentions. There are some cognitive differences between belief and reliance, but the relevant issue for us is their normative differences.

Reliance is tightly connected to interpersonal obligation, since inducing or reinforcing reliance is plausibly seen as a mechanism for creating obligation. What grounds this claim? Here again, we follow Scanlon, although employing a different one of his principles, *Principle L*. This principle states that we are obligated to take reasonable steps to prevent a significant loss that results from relying on expectations we have intentionally or negligently induced or reinforced.^38^ These reliance losses may include things like wasted time or resources, opportunity costs, and misspent energy.

Because attitudes of reliance are part of the constitutive base of collective intention for Alonso, we get a much different picture of the connection between the two aspects of collective intention - socio-psychological and normative - than we did in either Bratman or Roth. Dual-aspect views straddle the non-normativist/normativist distinction since they do not treat collective intention and interpersonal obligation as independent if at times overlapping or as essentially related phenomena. Instead, collective intentions normally bring interpersonal obligation with them, since part of their constitutive base involves an attitude that, under normal circumstances, generates such obligations when induced or reinforced.

This account explains the empirical research remarkably well. Not only does it generate obligations in cases in which *Principle F* doesn’t apply, since it doesn’t require explicit assurances, it also generates what look like the right obligations. Unlike *Principle F, Principle L* does not require the obligated person to receive release from the person to whom they are obligated. These are not obligations of strict performance; they may be satisfied by giving a timely warning instead. The obligation to give a timely warning grounded in the avoidance of reliance losses is a plausible philosophical explanation of the obligation to notify attested to by the participants in the studies, in part because it doesn't generate the stronger obligations of *Principle F*. Alonso’s dual-aspect view therefore provides us with a theory of collective intention that explains (i) the obligation to notify, without conflicting with (ii), the lack of an obligation to seek permission.

But there are two problems with the view as it stands, both stemming from Alonso’s specific grounding of the interpersonal obligations in this Scanlonian principle. The first results because *Principle L* states that the person who induces reliance must have reason to believe that the person in whom reliance is induced or reinforced will suffer significant loss as a result of their expectation being disappointed. It seems like not all collective actions involve sufficiently significant reliance losses if one of the participants doesn’t do their part.^39^ Remember that, as normally characterized, reliance losses may be in terms of energy, time, or resources. Consider again Bratman’s 5th avenue walking example, which was the basis of the experimental vignettes. The characters are independently walking down the street. They don’t expend any energy going out of their way, since they bump into each other. They don’t waste any time since no prior planning is involved in setting up the walk. And no extra resources are spent continuing a walk one is already on. So, it looks like there is a case of collective action that doesn’t involve reliance losses. Given this, the person who leaves doesn’t have reason to believe that the person who is left will suffer reliance losses. Without reason for this belief, there is no obligation to avoid such losses. So, in the 5th avenue walking case, there is no obligation to give a timely warning. Yet this is exactly the case in which the participants in the empirical research judge that there is such an obligation. So, at least in this case, Alonso’s theory does no better than other quasi-psychological views at explaining the experimental results.

The second issue is with the experiment result that has not yet been discussed, namely (iii), the obligation to notify in cases of morally wrong collective action. Alonso explicitly denies that such an obligation exists.^40^ About a case involving a collective intention to rob a bank, he claims that, “Since robbing a bank is a morally impermissible action, our having formed such a shared intention does not create relevant obligations between us”, and goes on two show how the socio-psychological structure is enough to play the roles of collective intention in these cases.^41^ Later he makes it explicit that he is relying on his own intuitions for this judgment about interpersonal obligation, stating that in these cases “it is intuitively clear that no obligations are created between the individuals”.^42^ The conflict with the experimental research on this issue is acute. The vignettes that make up the conditions in the studies leading to (iii) are on based on a case with many similarities to robbing a bank. Here again Alonso’s theory does no better than Bratman’s at explaining the results.

If we accept that a theory of collective intention should be sensitive to our social practices surrounding collective action, and that the experimental research gives us some purchase on those practices, there is reason to have a view that accounts for these two issues. So, despite the valuable advance made by Alonso’s dual aspect view, especially with respect to the relation between those aspects, there is further work to be done for quasi-psychologists.

## Conclusion

Some arguments for particular theories of collective intention and action in the philosophical literature in part rely on the arguer’s intuitional judgments about thought experiments. As such, one issue to which they may be sensitive is the extent to which those intuitions capture our common understanding of what’s required of us when we act together. This may be revealed by experimental research. Recently, there has been a turn to investigating these questions using empirical methods based on the thought experiments philosophers have used to support their views. In this paper, I’ve explored how a particular class of views, labelled “quasi-psychologism”, is able to account for this research.

The results so far give us initial reason to accept three claims about our common understanding of collective action: it involves an obligation to notify other participants when leaving, but not an obligation to seek their permission, and the obligation to notify is present even when the action is morally wrong. Explaining these results creates difficulties for extant quasi-psychological views, given the normative frameworks they employ and assumptions on which they’re built. This, however, does not speak decisively against them. Rather, on the assumption that it is a virtue of a theory of collective intention and action to be able to explain this research, the conclusion we should draw is that further normative theorizing is required to make sense of the interpersonal normativity in collective action within the metaphysical constraints of quasi-psychologism.

## Footnotes

1 I am assuming that constitution is not identity.

2 While not exhaustive, since other relations between individual psychological states and collective intentions are possible, this division captures all of the major proposals in the literature on collective intention and action. See the following notes for references. It does not include views that require the creation of a supra-individual center of agency, as is frequently the case in the literature on “group agency”.

3 Versions of this argument may be found in Bratman (1993), Velleman (1997), and Gilbert (2013), in response to the psychological view put forward by Searle (1990).

4 See Alonso (2016), Bratman (2014), and Roth (2014) for versions of this claim. The only major defender of non-psychologism is Gilbert (1990,  2009, 2013), although arguably the view put forward in Velleman (1997) qualifies, since he claims that shared intentions may be constituted by speech acts rather than mental states.

5 The terms “normativist” and “non-normativist” are used in roughly the same way in Alonso (2009) and Schmid (2009).

6 This paradigm is developed primarily in Gomez-Lavin & Rachar (2019) and Gomez-Lavin & Rachar (forthcoming). It is descriptive in the sense that it aims to understand the obligations people take themselves to have in certain contexts, rather than provide any justification for those judgments.

7 (2013, p. 102).

8 (2013, p. 24).

9 (2014, p. 86).

10 Bratman (2006, p. 7).

11 Gilbert (2013).

12 There is a clear difference between these cases in that in the Gilbert example they set out on a walk together, while in the Bratman example they simply bump into each other. However, Gilbert explicitly discusses this distinction and claims in makes no difference to the normative situation between the participants (2013, p. 24–26, especially fn. 6).

13 Another area of equally pronounced disagreement between the intuitions of Bratman and Gilbert concerns the non-psychological view’s claim that there can be shared intentions without corresponding individual intentions. See Gilbert (2013) and Bratman (2014) for the opposing intuitions.

14 Other considerations in favour of the use of experimental philosophy to extend and improve upon aspects of traditional philosophical methodology are advanced by proponents of the “positive program” in experimental philosophy. See, for example, Glasgow (2008) and Murray and Nahmias (2014). For a discussion of the positive program and how it relates to other interpretations of and aims for experimental philosophy, see Mallon (2016). I take the assumptions here to be in line with the positive program.

15 See Gomez-Lavin & Rachar (2019) for more details.

16 Gomez-Lavin & Rachar (2019).

17 See Gomez-Lavin & Rachar (forthcoming) for more details.

18 See Gomez-Lavin & Rachar (2019).

19 See Bratman (1999) for this quote and further characterization of his view.

20 For a list and discussion of the norms in question, see Bratman (2014).

21 Bratman (2009, p. 151–2).

22 The most relevant example is Bratman (1999).

23 One passage where Bratman uses this language is (1999, p. 126). In it, he is clear that he means “unreasonable” in a sense that does not grant one’s partner a special claim sufficient to ground an obligation.

24 This point about the directedness of the obligations is suggested by, but not yet fully developed in, the experimental research. Sometimes the questions in the research are framed in a way that suggests one character has a right against another, see for example the “Right to Rebuke” measure in experiment 3 in (Gomez-Lavin & Rachar, 2019), p. 105–108), but not in such a way that there is definitive evidence that the participants see all such relations as directed. Further research is the needed to support this point. However, that these obligations are directed is commonly assumed in the literature, see for example Gilbert (2013), Roth (2004,  2014), and Alonso (2009) and so I will proceed under that assumption here.

25 Bratman (2014).

26 (1999, 135).

27 See Scanlon (1998) for discussion.

28 Bratman (1999).

29 In fact, the “obligation to seek permission” is only a necessary part of what is required by *Principle F*. But as a necessary part, if it turns out it isn’t present, we may conclude that the stronger obligations generated by *Principle F* are not present.

30 Gilbert (1990).

31 The theory is put forward, and this label is used, in Roth (2004,  2014).

32 (2004, p. 383). See also, (2014, p. 642): “your intention figures in my practical thought in the way that my own intentions do,” when practical intimacy exists between us.

33 Roth (2004).

34 For more details about the difference between re-issuing and preserving, on the one hand, and executing, on the other, see Roth ( 2004).

35 (2004, p. 390).

36 For further discussion of this point, see Roth (2004) and (2014, p. 643–4).

37 His theory is developed primarily in his (2009, 2016).

38 For Scanlon’s account, see his (1998). For Alonso’s discussion of Scanlon, including his modification of *Principle L* to account for merely reinforcing reliance, see his (2016, p. 290, especially fn. 33).

39 This issue is acknowledged by Alonso (2009) and raised by Bratman as a concern for Alonso’s view (2014, p. 184, fn. 6), although not in relation either to the obligation to notify or the walking case.

40 See (2009, p. 472) and (2016, p. 291–2, 294–5) for Alonso’s main discussions of the topic.

41 (2016, p. 292).

42 (2016, p. 294).
